# Characterization of extended co-culture of non-typeable *Haemophilus influenzae* with primary human respiratory tissues

**DOI:** 10.1258/ebm.2012.011377

**Published:** 2012-05

**Authors:** Dabin Ren, Kevin L Nelson, Peter N Uchakin, Arnold L Smith, Xin-Xing Gu, Dayle A Daines

**Affiliations:** 1 Division of Basic Medical Sciences, Mercer University School of Medicine, 1550 College Street, Macon, GA 31207; 2 Seattle Children's Research Institute, 1900 Ninth Avenue, C9S-8, Seattle, WA 98101; 3 National Institute on Deafness and Other Communication Disorders, 5 Research Court, Rockville, MD 20850, USA

**Keywords:** NTHi, NHBE, co-culture, persistence

## Abstract

Non-typeable *Haemophilus influenzae* (NTHi) are human-adapted Gram-negative bacteria that comprise part of the normal flora of the human upper airway, but are also responsible for a number of mucosal infections such as otitis media and bronchitis. These infections often recur and can become chronic. To characterize the effect of long-term co-culture of NTHi with human tissues, we infected primary respiratory epithelial cells grown at the air–liquid interface with three NTHi strains over a range of 1–10 days. Scanning and transmission electron microscopy of tissues confirmed that intact NTHi were persisting paracellularly, while organisms observed in intracellular vacuoles appeared degraded. Furthermore, the apical surface and tight junctions of the infected tissues were undisturbed, with high transepithelial electrical resistances, while the basal cell layer displayed more junctional disorganization and wider intercellular spaces than the uninfected control tissues. Although the tissues elaborated the cytokine profile reported for NTHi-caused otitis media *in vivo*, there was little change in the dynamics of cytokine secretion over the time points tested. Finally, we report that NTHi strains released outer membrane vesicles (OMVs) during extended co-culture with the tissues, and show that these OMVs directly interact with host cell membranes.

## Introduction

Non-typeable *Haemophilus influenzae* (NTHi) are small fastidious Gram-negative organisms that have evolved to be obligate parasites that constitute part of the normal flora of the human upper airway. In addition to being commensals, these bacteria are implicated in mucosal infections, including otitis media and bronchitis.^1,2^ Such infections can be recurrent and resistant to antimicrobial therapy, often requiring sequential treatments and, in the case of chronic otitis media with effusion, surgical intervention. Lower respiratory tract infections such as pneumonia and exacerbations of chronic obstructive pulmonary disease have also been attributed to NTHi.^3,4^ Furthermore, there is evidence that NTHi can form biofilms on mucosal surfaces *in vivo*.^5^ Taken together, these characteristics suggest that this organism can adapt to a number of different lifestyles, depending upon its immediate microenvironment in the host as well as other factors.

The traditional method of investigating how a pathogen interacts with human cells has been to grow epithelial cells as a submerged monolayer *in vitro* and then challenge with the pathogen. Various specific attributes, such as bacterial adherence and cell entry, can be measured by harvesting and enumerating either the total cell-associated bacteria (those that were adherent and those that had been internalized) or only those that had survived after the monolayer had been treated with gentamicin.^6^ Since this antibiotic does not enter human cells during the course of the assay, only those bacteria that were inside the human cells prior to the gentamicin treatment can survive. Using this approach, early events in the interaction of NTHi with human cells can be efficiently and effectively quantified. However, since NTHi can also grow in the overlying cell culture media as well as enter the epithelial cells, this assay is limited in the amount of time that the monolayer can be co-cultured with the bacteria (usually hours) before unacceptable human cell toxicity results. In addition, epithelial cells do not differentiate or polarize well in a submerged culture.^7^ These issues were addressed by a subsequent model that consists of first growing primary epithelial cells on a porous membrane of a tissue culture insert, with media above and below the membrane. After the cells reach confluence, the apical media is removed. This raises the epithelial cells to the air–liquid interface (ALI) and allows the cells to be fed by media from the basal side.^8^ In this study, primary human respiratory epithelial cells at the ALI are allowed to differentiate and polarize for a number of days before being co-cultured with NTHi, which are inoculated directly onto the apical surface. Particularly when using primary human respiratory epithelial cells, this approach is both quite suitable for long-term co-culture with NTHi and is biologically relevant, as it is an excellent *in vitro* representation of human upper airway tissue.^7^ In addition, since the epithelium of the middle ear is part of the respiratory mucosa and produces, as do bronchial epithelial cells, a complex mixture of mucin and cytokines, this model is appropriate for the study of otitis media pathogens.^9^ We hypothesized that NTHi causes an acute inflammatory response upon contact, and investigated the effect of co-culture on the tissues. Herein, we present data on how and where NTHi survives over time within this ALI model, how the tissues react to long-term bacterial infection and the maintenance of transepithelial electrical resistance (TER) following NTHi co-culture with both normal human bronchial epithelial (NHBE) cells (Lonza, Walkersville, MD, USA) at the ALI, and a commercially available tissue model, the EpiAirway™ (MatTek, Ashland, MA, USA). The EpiAirway tissues consist of primary, well-differentiated, three-dimensional tissues with tight junctions, ciliated and non-ciliated cells, goblet cells that produce mucin, and which retain the ability to produce cytokines in response to infection.

## Materials and methods

### Bacterial cultures

All bacterial strains used in this study are pertinent to otitis media. Strain R2846 was isolated from the middle ear of a child with otitis media.^10^ Strain R2866 was isolated from the blood of a child with otitis media^11^ and strain 86-028NP was isolated from the nasopharynx of a child with otitis media.^12^ The genome of each of the strains has been sequenced and annotated, facilitating future comparative studies. NTHi strains were grown overnight at 37°C in 5% CO_2_ on chocolate agar plates with 5 U/mL bacitracin, or brain heart infusion agar supplemented with 10 *μ*g/mL of *β*-NAD and 10 *μ*g/mL of hemin chloride (sBHI). Broth cultures were grown in sBHI at 37°C with shaking in room air.

### ALI NHBE cell cultures

For NHBE cell (#CC-2540; Lonza) ALI cultures, 5.0–7.0 × 10^5^ NHBE cells were seeded and grown submerged on BD Biocoat membranes with a 4.2 cm^2^ growth area and a 3 *μ*m pore size in 50/50 bronchial epithelial cell basal medium (#CC-3171; Lonza)/Dulbecco's modified Eagle's medium (#10 013 CM; Mediatech cellgro, Manassas, VA, USA) with bronchial epithelial growth medium SingleQuots (#CC-4175; Lonza) containing retinoic acid (#R2625; Sigma, St Louis, MO, USA) at 10× the reference concentration^13^ until confluence was reached. This medium is similar to Matsui-2 described by Sachs *et al.* ^14^ and is hereinafter referred to as TC medium. At this time, the apical medium was removed and 2.5 mL of TC medium was added to the basal compartment. The TER was measured using EVOM2 (World Precision Instruments, Sarasota, FL, USA) according to the manufacturer's instructions. Between three and five days after being placed at ALI, the TER of the NHBE cells stabilized at ∼400 Ω cm^2^, which starts to decrease after several weeks at the ALI. The apical side of each insert was subsequently inoculated with ∼1.0 × 10^3^ colony-forming units (CFU) of NTHi strain R2846 in a volume of 200 *μ*L in Dulbecco's phosphate-buffered saline (D-PBS). Data are representative of a total of six different lots of NHBE cells.

A total of seven different lots of EpiAirway tissues (#AIR-100-ABF or #AIR-606-ABF; MatTek, Ashland, MA, USA) were grown on collagen-coated Millipore Millicell™ CM (Millipore, Billerica, MA, USA) single-well tissue culture plate inserts with a pore size of 0.4 *μ*m and received 22 days after seeding. After at least 24 h of equilibration at 37°C in 5% CO_2_, each insert was inoculated with NTHi strains R2846, R2866 or 86-028NP. The initial TER of each tissue was ∼250 Ω cm^2^. The proprietary basal medium (#AIR-100-MM; MatTek) was changed and the apical surfaces were washed with 200 *μ*L of D-PBS on a daily basis.

### ALI co-cultures

Overnight cultures of NTHi strains were resuspended from chocolate or sBHI agar plates into D-PBS at an optical density (600 nm) of ∼0.2. These initial solutions were diluted in D-PBS to the desired concentrations (∼1.0 × 10^3^–1.0 × 10^7^ CFU) and used to apically inoculate ALI NHBE cells or EpiAirway tissues. The 0.6 cm^2^ growth area inserts were inoculated with ≤25 *μ*L, while the 4.2 cm^2^ inserts received 200 *μ*L of the bacterial suspensions.

### ALI harvest

At the desired time points, each insert was washed 3× with D-PBS, and incubated both apically and basally with TC or basal media containing 100 *μ*g/mL gentamicin for one hour at 37°C. The inserts were washed 3× with D-PBS without calcium and magnesium, and then incubated for 10 min at 37°C with 1% saponin in D-PBS without calcium and magnesium. Following this, each insert was scraped and the tissues were mechanically disintegrated, diluted and plated for viable bacteria as described previously.^15^ The surviving organisms were expressed as gentamicin-resistant CFU/cm^2^ of the growth area.

### Enzyme-linked immunosorbent assay assays

EpiAirway washes were first analyzed with the Multi-Analyte ELISArray kits (SABioscienses, QIAGEN Inc, Valencia, CA, USA) for exploratory detection of cytokines. Results were read on a Tecan Infinite^®^ M1000 Plate Reader (Tecan Group Ltd, Männedorf, Switzerland) at 450 nm with *λ* correction of 570 nm followed by acquisition and reduction with data analysis software Magellan (Tecan Group Ltd) for consequent statistical analysis. Subsequently, electrochemiluminescence detection in multi-plex format (Meso Scale Discovery, Gaithersburg, MA, USA) was used for cytokine quantification. Results were read on a MSD Sector Imager 2400E followed by acquisition and reduction with data analysis software Discovery Workbench 3.0.18 (Meso Scale Discovery).

### Statistical analysis

All graphical data are expressed as the mean ± SD of the indicated number of experiments. The level of significance was set at *P* < 0.05. The statistical software package SigmaStat (Systat Software Inc, Chicago, IL, USA) was used for statistical analysis of the collected enzyme-linked immunosorbent assay (ELISA) data. Kruskal–Wallis one-way analysis of variance on Ranks was implemented to determine statistical differences between studied intervals of sample collection for each cytokine.

### Immunoelectron microscopy

EpiAirway inserts were first co-cultured as described above with NTHi strains R2866 or 86-028NP for 5 days prior to fixation, or with D-PBS sham inoculation. The inserts were then washed and immersed in 4% paraformaldehyde and 0.2% glutaraldehyde in 100 mmol/L sodium cacodylate buffer (pH 7.4) for one hour at room temperature, followed by 18 h at 4°C. Each membrane was then removed from the plastic insert using a #11 pointed scalpel blade, immersed in fixative and processed by the Georgia Health Sciences University (GHSU) Electron Microscopy Core (Augusta, GA, USA). After incubation with anti-NTHi primary antibody followed by gold-labeled secondary antibody, the grids were stained briefly with uranyl acetate and imaged using a JEOL JEM 1230 transmission electron microscope (TEM; JEOL USA Inc, Peabody, MA, USA).

### Transmission and scanning electron microscopy

Images of infected and uninfected (control) EpiAirway inserts were obtained after fixation in 1.25% glutaraldehyde, 2.0% paraformaldehyde in 100 mmol/L sodium cacodylate buffer (pH 7.2) for 24 h. The membranes were then removed from the plastic insert using a #11 pointed scalpel blade, immersed in fixative and processed by the GHSU Electron Microscopy Core. Briefly, the fixed membranes were osmicated^16^ and cut in half. The TEM half was dehydrated, infiltrated with LR White, sectioned and imaged using a JEOL JEM 1230 transmission electron microscope. The scanning electron microscopy (SEM) half was critical-point dried with CO_2_, adhered to stubs and sputter-coated with gold–palladium prior to imaging on a FEI XL30 field emission gun SEM (FEI Company, Hillsboro, OR, USA). This resulted in paired SEM and TEM images of the same insert.

## Results

### NTHi strains survive within ALI tissues for extended periods of time

**Figure 1:**
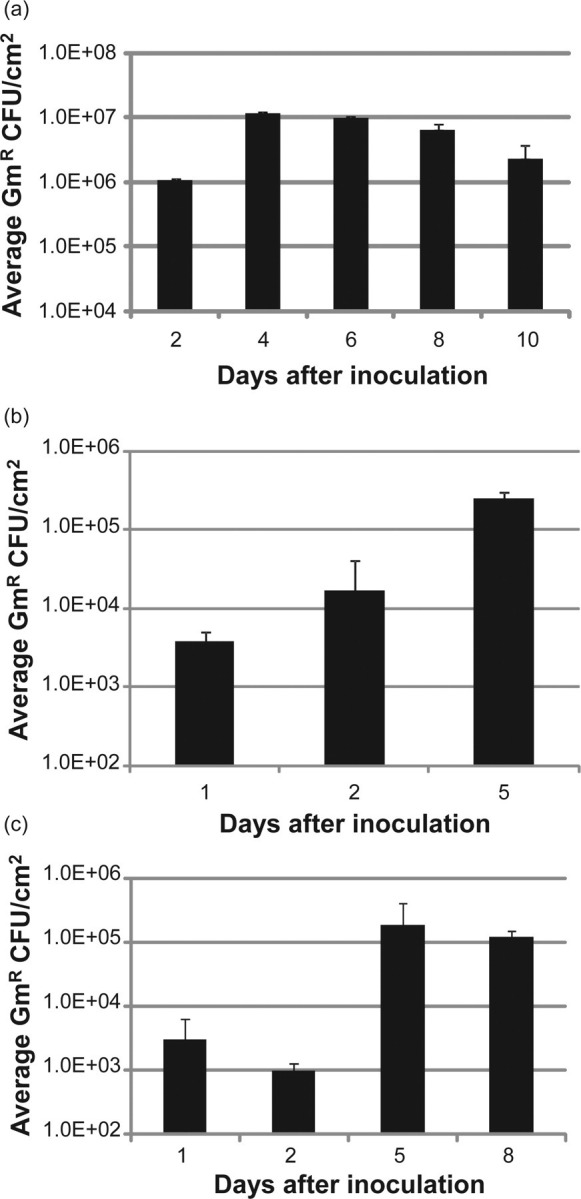
Survival of NTHi strains over time inside EpiAirway tissues and ALI NHBE cells. (a) Internalized (gentamicin-resistant) bacteria of strain 86-028NP recovered from EpiAirway tissues, 2–10 days after infection (*n* = 6). (b) Internalized strain R2846 recovered from ALI NHBE cells, 1–5 days after infection (*n* = 2). (c) Internalized strain R2846 recovered from EpiAirway tissues, 1–8 days after infection (*n* = 2). Error bars are SD. ALI, air–liquid interface; NHBE, normal human bronchial epithelial; NTHi, non-typeable *Haemophilus influenzae*

To determine the length of time that NTHi could be co-cultured with ALI tissues, we tested the ability of various NTHi strains to survive within ALI NHBE cells and EpiAirway tissues over time points ranging from 1 to 10 days (Figure 1). At each desired time point, the tissues were harvested and the gentamicin-resistant (internalized) bacteria were enumerated by plate counts. Figure 1a shows the numbers of bacteria surviving within EpiAirway tissues from long-term co-cultures, expressed as viable gentamicin-resistant CFU recovered per square centimeter of tissue (Gm^R^ CFU/cm^2^), for NTHi strain 86-028NP. These data are consistent with previous results observed for strain R2866.^15^ Figure 1b illustrates the Gm^R^ CFU/cm^2^ of strain R2846 in ALI cultures of NHBE cells, and Figure 1c shows R2846 recovered from EpiAirway tissues. This confirms that ALI cultures can be used to investigate the long-term interactions of various strains of NTHi with primary human epithelial cells *in vitro*.

### Long-term co-culture of NTHi with primary tissues at the ALI does not result in significant damage to the apical cell layer

**Figure 2:**
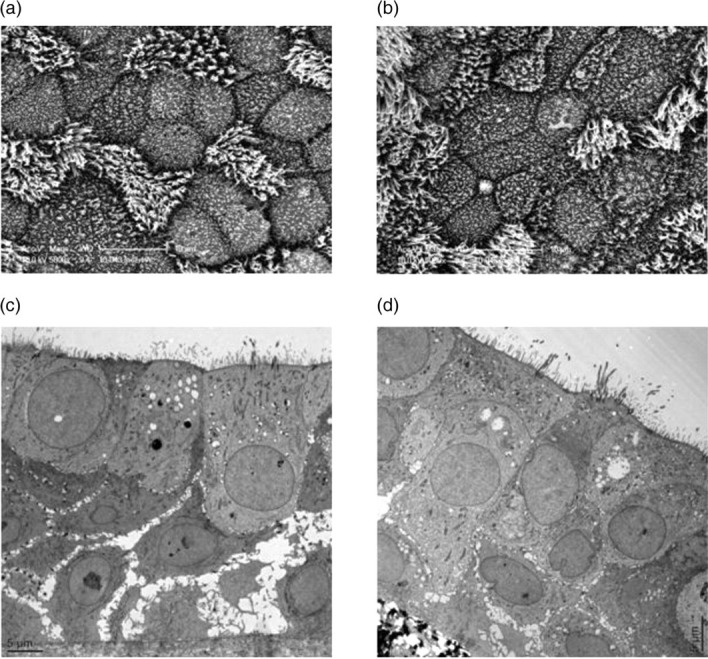
Paired scanning electron microscopy (SEM) and transmission electron microscopy (TEM) images of EpiAirway tissues with or without NTHi co-culture. One EpiAirway insert was infected with strain R2866 for five days, then fixed and cut in half. One half was processed for SEM (a), the other for TEM (c). Another EpiAirway insert was not infected with NTHi and used as the control for SEM (b) and TEM (d). (a) SEM of infected EpiAirway tissue. (b) SEM of control EpiAirway tissue. (c) TEM of infected EpiAirway tissue. (d) TEM of control EpiAirway tissue. NTHi, non-typeable *Haemophilus influenzae*

To examine the effect of extended NTHi co-culture on EpiAirway tissues, SEM and TEM images were taken of inserts with and without infection (Figure 2). These SEM and TEM images are paired, as each EpiAirway tissue was divided, with one half processed for SEM and the other half processed for TEM. Figure 2a shows a representative SEM image of tissue infected with strain R2866 for five days, while Figure 2b shows a SEM of the uninfected control EpiAirway tissue. There was no apparent difference in the number of ciliated cells or the length and number of microvilli between the infected and control tissues. However, there appeared to be more goblet cells in the infected tissues than in the control, with 70 goblet cells observed in 10 random SEM images of infected tissues, versus 46 identified in the same number of random images of uninfected tissues (each at ×5000 magnification).

To image any internal effects of NTHi co-culture on EpiAirway tissues, TEM was performed on each sample. Figure 2c shows a representative TEM image of tissue infected for five days with strain R2866, and Figure 2d shows the uninfected control tissue. Consistent with the SEM results, cells at the apical surface of the infected tissues were undamaged, with tight junctions and adherens junctions that appeared intact. However, the basal cell layer of the infected tissue exhibited more disorganized junctions, with wider intercellular spaces, than those in the control tissue.

### Infected tissues maintain polarization during extended periods of co-culture

**Figure 3:**
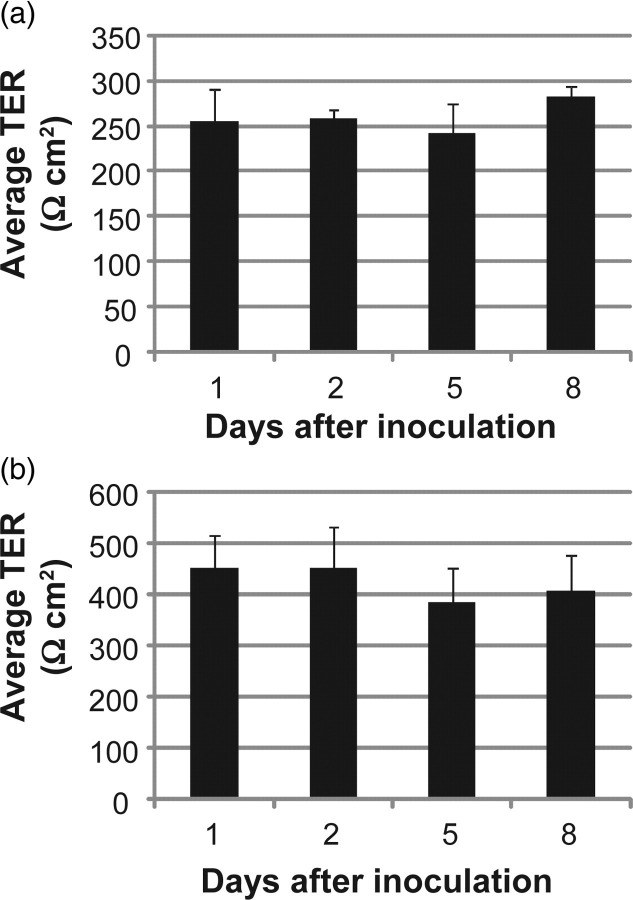
Transepithelial electrical resistance (TER) of EpiAirway (a) and ALI NHBE (b) cell cultures. (a) EpiAirway cultures were inoculated with strain R2846 and the TER was measured during co-culture. (b) NHBE cell cultures at the ALI were inoculated with strain R2846 and the TER was measured during co-culture. Horizontal axes denote time after inoculation for each reading. Error bars are SD. ALI, air–liquid interface; NHBE, normal human bronchial epithelial

After infection, the TER, a measure of functional tight junctions, was quantified over time in both EpiAirway tissues (Figure 3a) and ALI NHBE cells (Figure 3b) infected with strain R2846. Average TER values expressed in Ω cm^2^ are reported. In both models, no significant decrease in TER was observed over the course of the co-culture with NTHi. In a separate experiment, the TER of two ALI NHBE cell cultures was measured prior to infection with strain R2846, and again eight days after inoculation. To disrupt the TER in these infected ALI NHBE cell cultures, 0.2 mL of 0.02% ethylene glycol tetraacetic acid was added to the apical surface of each insert for four hours. The TERs decreased from more than 400 to 20 and 22 Ω cm^2^, respectively, indicating that the measured electrical resistance was due to functional tight junctions. These data support the SEM and TEM findings and confirm that extended co-culture with NTHi does not disturb epithelial cell tight junction integrity.

### Infected tissues elaborate proinflammatory cytokines that match those reported during *in vivo* infections

**Figure 4:**
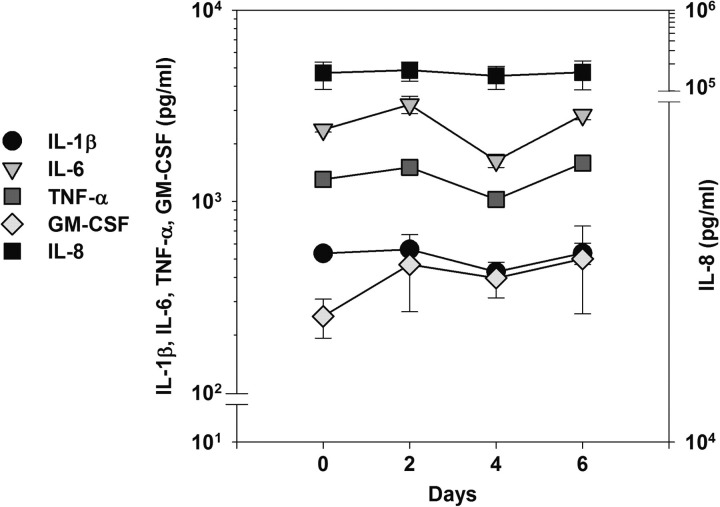
Cytokine profile of EpiAirway inserts infected with strain 86-028NP over time. ELISA assays were performed to detect the levels of IL-1*β*, IL-6, TNF-*α*, GM-CSF and IL-8 in pooled EpiAirway washes (*n* = 2) during co-culture. Day 0 indicates assays prior to inoculation, and the following days indicate length of time of co-culture. IL, interleukin; GM-CSF, granulocyte/macrophage colony-stimulating factor; ELISA, enzyme-linked immunosorbent assay; TNF, tumor necrosis factor

To determine whether the ALI tissues expressed the epithelial cell cytokine profile during long-term infections with NTHi *in vitro* that has been reported during infections *in vivo*,^17^ ELISA assays were performed on the pooled apical washes of EpiAirway tissues co-cultured with strain 86-028NP at day 0 (prior to infection) and at two, four and six days after infection (Figure 4). Interleukin-8 (IL-8) was the predominant cytokine, followed by IL-6, IL-1 and granulocyte/macrophage colony-stimulating factor (GM-CSF). Uninfected EpiAirway tissues produced approximately 200 ng/mL of IL-8 (Figure 4, day 0 time point), as airway epithelium produced IL-8 on a constitutive basis.^18^ No significant amounts of IL-2, IL-4, IL-10, IL-12p40, IL-17 or interferon-*γ* were detected in the samples. Further, there were no statistically significant changes in the dynamics of the secretion of tumor necrosis factor-*α*, IL-1*β*, IL-6, IL-8, and GM-CSF in the apical washes over the time points tested. This is consistent with a previous study showing that the mRNA levels of proinflammatory cytokines elicited by NTHi infections *in vivo* peaked at 3–6 hours after infection, and prior to the first clinical signs of otitis media.^19^

In a separate set of experiments using NHBE cells at the ALI, we assayed both the apical and basal compartments for the presence of IL-8 by ELISA at one day and eight days after inoculation with strain R2846. At one day, the average concentration of IL-8 in the apical washes was 251 ng/mL, while the concentration in the basal media was 69 ng/mL. At eight days, the average IL-8 concentration was 350 ng/mL apically and 120 ng/mL in the basal media. Thus, the basal compartment concentration of IL-8 was approximately 28% to 34% of the apical values in this system.

### NTHi long-term co-culture with EpiAirway tissues results in focal areas of infection

**Figure 5:**
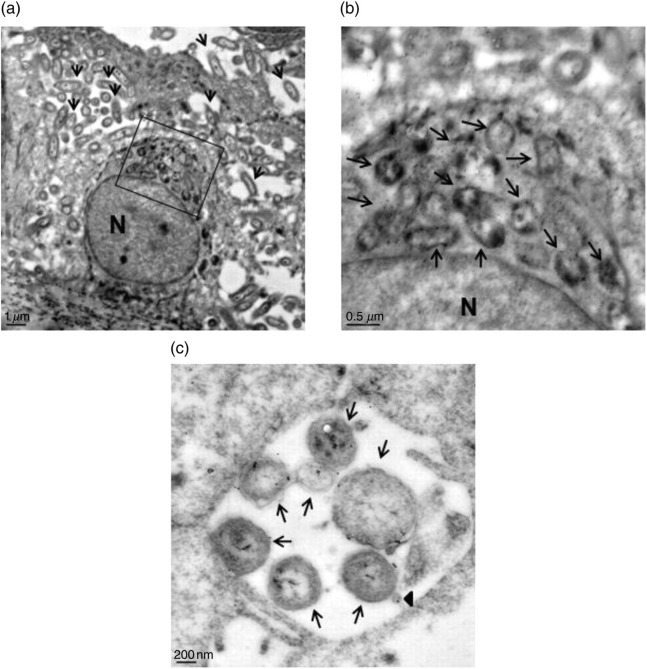
Immunoelectron microscopy of EpiAirway tissues co-cultured for five days with strains 86-028NP or R2866. Following co-culture, most of the intact 86-028NP are paracellular (a, arrows). The bacteria that are intracellular appear to be degraded (boxed area). N = nucleus. (b) Magnified view of the boxed area in (a). Arrows indicate the intracellular and degraded bacteria, which react with the anti-NTHi antiserum. N = nucleus. (c) Paracellular organisms of strain R2866. Arrows indicate intact bacteria; arrowhead indicates an outer membrane vesicle (OMV) that reacts with anti-NTHi antiserum. NTHi, non-typeable *Haemophilus influenzae*

Immunoelectron microscopy (IEM) with anti-NTHi antiserum followed by gold-labeled secondary antibodies of EpiAirway tissues after five days of infection with strains R2866 and 86-028NP demonstrated that NTHi were located in focal sites within the EpiAirway tissues, rather than existing as a uniform infection throughout the tissues (approximately 80 organisms are located between the cells shown in Figure 5a). The organisms observed within intracellular membrane-bound vacuoles appeared to be degraded, and IEM confirmed that these vacuoles contained NTHi (approximately 21 vacuoles reacted with the anti-NTHi antiserum shown in Figure 5b). The majority of the intact organisms visualized were located between the epithelial cells in the basal cell layers (Figure 5c). The paracellular location of NTHi following infection of immortalized human respiratory epithelial cells has been observed previously,^20^ and the presence of NTHi within intracellular vacuoles in primary cultures has also been reported.^21^ However, the duration of the co-culture of NTHi with the human cells for both of these studies was 30 h or less. Our data reveal that, over extended periods of co-culture with these ALI tissues, the location of intact NTHi is primarily paracellular, with intracellular vacuoles containing organisms in various stages of degradation.

### NTHi release outer membrane vesicles within the EpiAirway tissues

**Figure 6:**
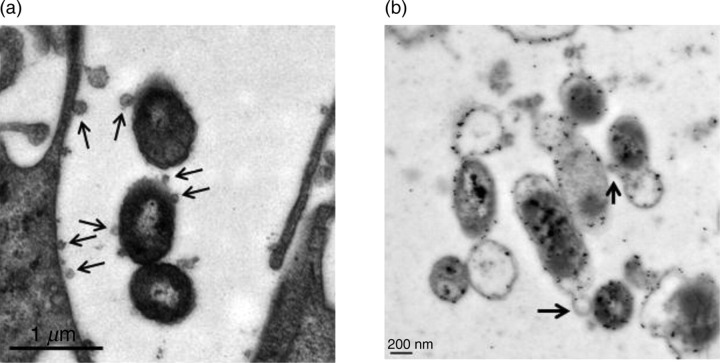
NTHi release outer membrane vesicles while co-cultured with EpiAirway tissues. (a) TEM of strain R2866 after a five-day co-culture with EpiAirway tissues. The bacteria are paracellular and are releasing OMVs (arrows), some of which appear to interact with the host cell membrane. (b) IEM which shows strain 86-028NP co-cultured with EpiAirway tissues for five days releasing OMVs that react with anti-NTHi antiserum (arrows) while located paracellularly. NTHi, non-typeable *Haemophilus influenzae*; TEM, transmission electron microscopy; IEM, immunoelectron microscopy

We observed that both strains R2866 and 86-028NP released outer membrane vesicles (OMVs) during long-term co-culture with EpiAirway tissues (Figure 6). The vesicles appeared to be shed when the organisms were paracellular, with some vesicles associating with the lateral membrane of the epithelial cells (Figure 6a). These OMVs were labeled with anti-NTHi antiserum and were identified by IEM, indicating that they originated from the NTHi strains (Figure 5c, arrowhead; Figure 6b, arrows). The role of these OMVs during long-term infection is currently under investigation.

## Discussion

The normal lifestyle of NTHi is as a human commensal, and its role as a pathogen can be considered to be accidental or opportunistic. Due to these unique characteristics, investigators are interested in determining how NTHi interact with respiratory epithelial cells, the site of colonization by *H. influenzae*, which can lead to superficial or invasive infection as a result of this interaction. Many important discoveries regarding NTHi pathogenic mechanisms and interactions with host cells have been made using submerged monolayer models,^22–24^ but their utility for long-term co-culture is arguably less than using a differentiated tissue model at the ALI. Understanding how NTHi survive during chronic mucosal infections, particularly otitis media and bronchitis, necessitates the ability to observe the organisms interacting with and persisting within host tissues over extended periods of time.

Although an immortalized cell line can be used to form the basis of the ALI model, primary human respiratory epithelial cells are particularly well suited for the study of NTHi infections of days or weeks duration. The detection of the major proinflammatory cytokines of the innate immune response during co-culture with NTHi suggests the adequate immune competence of the cells composing the tissues, even though many elements of the innate immune response and all of the adaptive are not present. This model allows investigators to study the interaction of NTHi within host tissues without the requirement to obtain Institutional Review Board approval, as the investigator is blinded to donor information by the company supplying the tissues and/or primary cells. Primary cells can also be pooled, decreasing the possibility of single-donor genetic factors influencing a particular lot of tissues. Finally, these ALI tissues are ideal for studying the long-term host–pathogen interactions, particularly for the *in vitro* modeling of recurrent diseases such as otitis media and lung infections.

It is known based on clinical observations in which children had nasopharyngeal colonization without signs or symptoms of inflammation that NTHi strains can interact with host cells for months without eliciting an appreciable inflammatory response,^25^ and these organisms have been observed growing as biofilms on mucosal surfaces *in vivo.* ^5^ However, it has also been well documented that shortly after inoculation, NTHi strains adhere to, and invade, host cells *in vitro.* ^6,20,21,26^ This paradox suggests that there are mechanisms by which NTHi can survive within the tissues over time without overtly damaging the epithelium and triggering host defenses. Our data, although limited to days rather than months, indicate that NTHi strains form focal communities within tissues at the ALI that do not disturb the structure and function of the apical cell layer or the TER of the tissues, and result in low levels of epithelial cell proinflammatory cytokine expression. This lifestyle makes sense for an organism whose survival depends upon its ability to scavenge essential nutrients without initiating its own destruction by the host response.

We also report here that NTHi release OMVs while persisting inside host tissues. Most Gram-negative bacteria shed these vesicles, both constitutively during normal growth, and to a greater extent under stressful environmental conditions.^27^ OMVs from various organisms have been shown to contain lipopolysaccharide and proteins as well as DNA,^28,29^ and a recent study has identified the proteome of OMVs shed from NTHi during stationary phase growth in rich media *in vitro*.^30^ A number of reasons for OMV shedding have been proposed, including niche competition with other bacteria, as a mechanism of horizontal gene transfer, as a method of antigen delivery to host cells, and as quorum-sensing molecule shuttles in biofilms.^31,32^ Our data demonstrate that NTHi shed these OMVs during long-term infections within primary human respiratory tissues at the ALI and confirm that the vesicles can, and do, interact with host cell membranes in this model.

We conclude that the results of this study provide direct and novel evidence of how NTHi interact with, and survive within, host tissues over time. During long-term co-culture with primary human respiratory tissues at the ALI, NTHi strains do not elicit a significant inflammatory response, as measured by a substantial increase in cytokine release, or damage the apical surfaces. Further, TER measurements confirm that extended infection with NTHi does not interfere with, or disrupt, junctional integrity. Interestingly, we found that the organisms survived within the tissues in paracellular foci of infection, with those observed located in intracellular vacuoles appearing to be degraded. Finally, we showed that NTHi release OMVs during long-term infection in this model, and that these vesicles can bind to host cell membranes. Experiments are ongoing to determine the role of these vesicles during NTHi infections.

**Author contributions:** All authors participated in the design, interpretation of the data and review of the manuscript. DR, KLN, PNU and DAD performed the experiments; X-XG provided critical reagents (*α*-NTHi antibodies); and DAD, PNU and ALS wrote the manuscript.
